# Improved accuracy of intraocular lens power calculation by preoperative management of dry eye disease

**DOI:** 10.1186/s12886-021-02129-5

**Published:** 2021-10-13

**Authors:** Jinsoo Kim, Mee Kum Kim, Yuseung Ha, Hae Jung Paik, Dong Hyun Kim

**Affiliations:** 1 Department of Ophthalmology, Gil Medical Center, Gachon University College of Medicine, 21, Namdong 774 beon-gil, Namdong-gu, Incheon, 21565 South Korea; 2 Department of Ophthalmology, Seoul National University College of Medicine, Seoul, Korea; 3 RudaCure Co., LTD, Incheon, Korea

**Keywords:** Dry eye, Cataract surgery, Intraocular lens power calculation, Preoperative treatment

## Abstract

**Background:**

To evaluate the effects of pretreatment for dry eye disease (DED) on the accuracy of intraocular lens (IOL) power calculation.

**Methods:**

Patients who underwent uneventful cataract surgery were included in the study. IOL power was determined using the SRK/T and Barrett Universal II (Barrett) formulas. The patients were divided into non-pretreatment and pretreatment groups, and those in the pretreatment group were treated with topical 0.5% loteprednol etabonate and 0.05% cyclosporin A for 2 weeks prior to cataract surgery. Ocular biometry was performed in all groups within 2 days before surgery. The mean prediction error, mean absolute error (MAE), and proportions of refractive surprise were compared between the non-pretreatment and pretreatment groups at 1 month postoperatively. Refractive surprise was defined as MAE ≥ 0.75D.

**Results:**

In a total of 105 patients, 52 (52 eyes) were in the non-pretreatment group and 53 (53 eyes) in the pretreatment group. The MAE was 0.42 ± 0.33, 0.38 ± 0.34 (SRK/T, Barrett) and 0.23 ± 0.19, 0.24 ± 0.19 in the non-pretreatment and pretreatment groups, respectively (*p* < 0.001/=0.008). The number of refractive surprises was also significantly lower in the pretreatment group. [non-pretreatment/pretreatment: 9/2 (SRK/T); 8/1 (Barrett); *p* = 0.024/0.016]. Pretreatment of DED was related to a reduction in postoperative refractive surprise. [SRK/T/Barrett: OR = 0.18/0.17 (95% CI: 0.05–0.71/0.05–0.60), *p* = 0.014/0.006].

**Conclusions:**

The accuracy of IOL power prediction can be increased by actively treating DED prior to cataract surgery.

## Introduction

The number of cataract surgeries has grown significantly in recent years due to an increase in lifespan in the elderly population [1]. There is no doubt that achieving a clear and precise vision after cataract surgery is greatly related to the quality of life of patients; hence, the factors that contribute to improved outcomes must be proactively addressed. Diverse premium intraocular lenses (IOLs), such as multifocal and toric IOLs, have been introduced. These IOLs are chosen according to the patients’ practical needs and ocular conditions. Hence, the accuracy of IOL power calculation is more emphasized now than it was in the past [2–4].

Dry eye disease (DED) is one of the most common ocular diseases affecting millions of people worldwide. It is mainly associated with tear film instability, tear hyperosmolarity, and inflammation of the ocular surface [5, 6]. It is also associated with foreign body sensation, occasional significant pain, and a decrease in quality of life [7]. Many studies have shown a negative impact of DED in cataract surgery. Patients who are predisposed to DED frequently express aggravated symptoms of DED after cataract surgery, which often results in less satisfactory visual outcomes [8, 9]. According to the preoperative treatment algorithm for ocular surface disorders from the recent clinical committee of the American Society of Cataract and Refractive Surgery, treatment of DED prior to cataract surgery can optimize surgical outcomes and patient satisfaction [10]. Additionally, it is important because DED can increase the variability in preoperative anterior corneal power measure, leading to inaccurate IOL power prediction [11, 12].

Hovanesian et al. showed that treatment with lifitegrast for 1 month prior to cataract surgery increased the accuracy of IOL power prediction, thereby highlighting the importance of preoperative treatment of DED [10, 12]. In addition, we have recently shown the excellent effects of short-term corticosteroid therapy in refractive DED patients [13]. In this study, we investigated whether short-term pretreatment of DED using topical loteprednol etabonate (LE) and cyclosporin A (CsA) before cataract surgery improves the accuracy of IOL power calculation.

## Methods

This retrospective observational study was conducted in accordance with the tenets of the Declaration of Helsinki, and ethical approval was obtained from the Institutional Review Board of Gachon University Gil Medical Center, Korea (IRB number: GCIRB 2021–170). Patients who underwent uneventful cataract surgeries between January 2018 and May 2020 were enrolled in this study. Exclusion criteria were patients with corneal opacity, history of corneal refractive surgery, intraoperative posterior capsular rent, and a history of ocular trauma affecting zonular damage. Patients who were unable to calculate IOL power using IOLMaster 500 (Carl Zeiss Meditec, Jena Germany) due to severe cataract or poor cooperation were also excluded. When the SNR value was greater than 2 and the standard deviation of the K value was less than 0.01D, the ocular biometry was measured once with IOLMaster500. The right eye was enrolled if the patient underwent cataract surgery in both eyes.

**Fig. 1:**
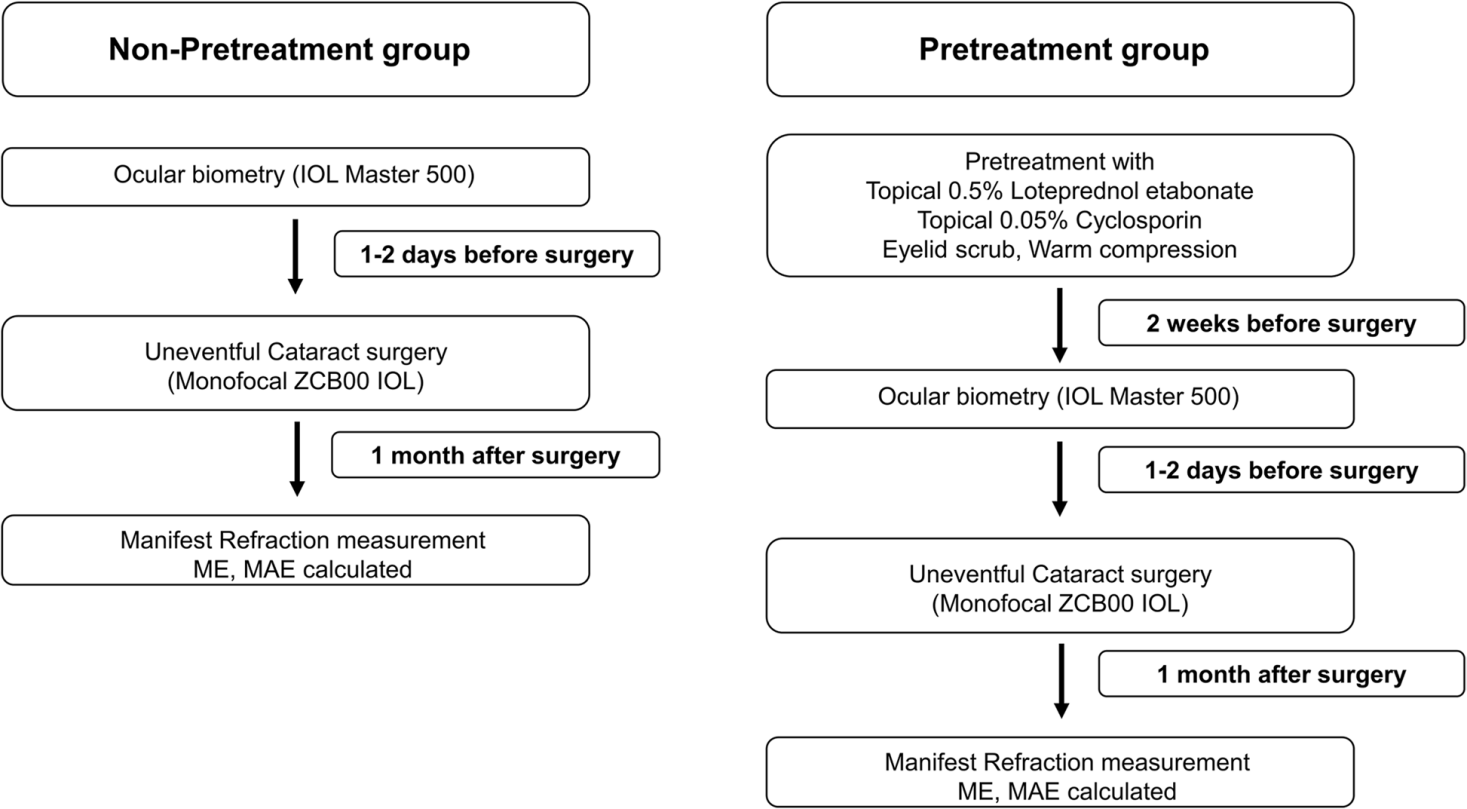
Schematic outline of the study. (ME: mean error; MAE: mean absolute error)

To manage preoperative DED, patients in the pretreatment group were administered 0.5% LE (Lotepro; Hanlim Pharm. Co.,Ltd) four times a day and 0.05% CsA (Tsporin; Hanlim Pharm. Co., Ltd) twice a day for 2 weeks prior to surgery (Fig. 1). Eyelid scrub and warm compression were also recommended for preoperative DED management. Patients in the non-pretreatment group did not use any eye drops except for the usual artificial tear before surgery. The management protocol for pretreatment of DED was started in January 2019, and patients who had undergone cataract surgery between January 2018 and January 2019 did not receive any pretreatment for DED. Cataract grading was assessed using slit lamp examination according to the Lens Opacities Classification System III standards under pupil dilation.

All cataract surgeries (phacoemulsification and posterior chamber lens implantation) were performed by a single surgeon (DH Kim) under topical anesthesia, with a superior corneal incision of 2.2 mm. A one-piece aspheric monofocal IOL (Tecnis ZCB00, Johnson & Johnson Surgical Vision) was used in all surgeries. The A-constant in SRK/T formula was 119.3 for Tecnis ZCB00, and this A-constant of ZCB00 was optimized from previous 500 operated eyes by DH Kim. Optimized A-constant were applied in both the non-pretreatment and pretreatment groups. When calculating the IOL power using the Barrett Universal II formula, the recommended A-constant of 119.39 in APACRS web was used. Ocular biometry was performed within 2 days before surgery in all groups.

Manifest refraction was measured 1 month after surgery, and the spherical equivalent (SE) was calculated. Prediction error was calculated by subtracting the preoperative predicted IOL power using the SRK/T and Barrett Universal II formulas from the postoperative actual SE. The absolute value of prediction error was used to prevent mathematical errors. The mean error (ME) and mean absolute error (MAE) of each formula were compared between the non-pretreatment and pretreatment groups. MAE was categorized into four range groups: MAE < 0.25D, 0.25D ≤ MAE < 0.5D, 0.5D ≤ MAE < 0.75D, and 0.75D ≤ MAE. Postoperative refractive surprise was defined as an MAE ≥ 0.75 D. Other clinical variables such as age, sex, axial length (AL), and keratometric (K) values were considered together to assess the effects of DED pretreatment on the accuracy of IOL power. Statistical analysis was performed using the SPSS Statistics (v. 18.0, SPSS Inc.). Independent t-test, chi-squared test, and logistic regression were used for analyses. Statistical significance was defined as a *p*-value of < 0.05. We compared the accuracy of intraocular power calculation between the non-pretreatment and pretreatment group for DED before cataract surgery, and analyzed which factors influence on the refractive surprise.

## Results

A total of 105 eyes of 105 patients were analyzed. The mean age of the patients was 67.9 ± 11.8 years. Among the 105 patients, 53 (53 eyes) received pretreatment for DED while 52 (52 eyes) did not. The baseline characteristics of the patients are summarized in Table 1. There were no differences in the AL, average K value, anterior chamber depth, tear film breakup time, ocular surface staining score, and tear secretion with the Schirmer test between the non-pretreatment and pretreatment groups (each *p* > 0.05, Table 1).

**Table 1:** Baseline characteristics in the enrolled patients

	Non-pretreatment group (*n* = 52 eyes)	Pretreatment group (*n* = 53 eyes)	*P* value
Age	67.7 ± 12.8	67.5 ± 10.8	0.924
Sex	M/F: 20/32	M/F: 18/35	0.687
Nuclear sclerosis grading	2.3 ± 0.9	2.5 ± 0.8	0.197
AL (mm)	23.74 ± 1.40	23.70 ± 1.14	0.874
Kavg (D)	44.49 ± 1.56	44.23 ± 1.50	0.377
ACD (mm)	3.06 ± 0.45	3.11 ± 0.35	0.257
TBUT (sec)	4.9 ± 1.8	4.6 ± 0.9	0.457
OSS (point)	0.1 ± 0.4	0.2 ± 0.55	0.381
Tear secretion (mm)	9.3 ± 2.8	8.2 ± 2.7	0.130

Values are presented as mean ± standard deviation

*ACD* Anterior chamber depth, *AL* Axial length, *Kavg* Mean keratometry value, *OSS* Ocular surface staining score according to the Oxford scale, *TBUT* Tear film breakup time

**Fig. 2:**
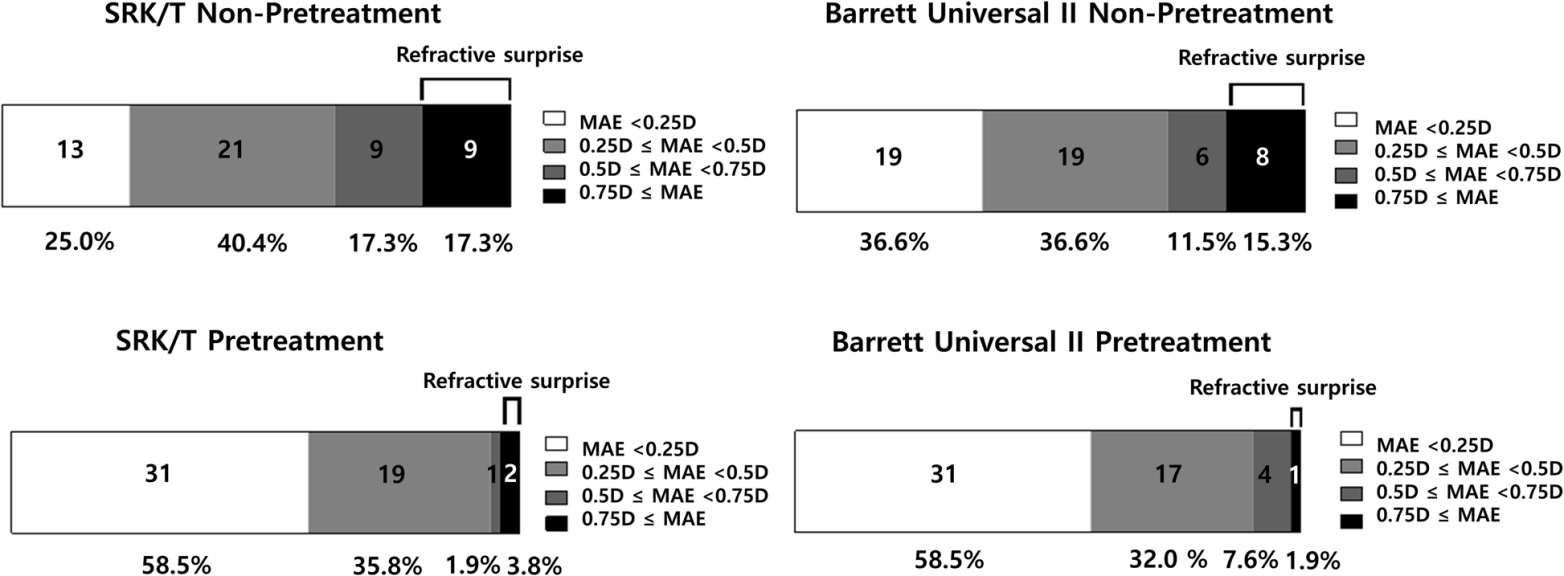
Stacked histogram comparing the percentage of cases within a given diopter range of predicted spherical equivalent refraction outcome between the DED non-pretreatment and pretreatment groups. (DED: dry eye disease; ME: mean error; MAE: mean absolute error; D: diopter)

The ME at 1 month after surgery was 0.10 ± 0.53D, − 0.06 ± 0.51D (SRK/T, Barrett universal II) in the non-pretreatment group and 0.09 ± 0.28D, − 0.01 ± 0.30D in the pretreatment group. There were no significant differences between the two groups (*p* = 0.828/0.529, Table 2). However, MAE at 1 month after surgery was 0.42 ± 0.33D, 0.38 ± 0.34D (SRK/T, Barrett universal II) in the non-pretreatment group and 0.23 ± 0.19D, 0.24 ± 0.19D in the pretreatment group. MAE in the pretreatment group was significantly lower than that in the non-pretreatment group (*p* < 0.001/=0.008, Table 2). The number of refractive surprises was also significantly smaller in the pretreatment group than in the non-pretreatment group [non-pretreatment/pretreatment: 9/2 (SRK/T); 8/1 (Barrett Universal II); *p* = 0.024/0.016; Table 2]. Figure 2 shows the stacked histogram comparing the number and percentage of cases within a given diopter range of predicted SE refraction outcome between the two groups. The pretreatment group showed a tendency of higher percentage of MAE ≤ 0.25D (58.5%/58.5%) and MAE ≤ 0.5D (94.3%/90.5%) than MAE ≤ 0.25D (25.0%/36.6%) and MAE ≤ 0.5D(65.4%/73.2%) of the non-pretreatment group for SRK/T and Barrett Universal II (MAE ≤ 0.25D; *p* = 0.001/0.020 and MAE ≤ 0.5D; *p* = 0.002/0.154 for SRK/T and Barrett Universal II, respectively).

**Table 2:** Comparison of the accuracy of intraocular power calculation between the non-pretreatment and pretreatment group for DED before cataract surgery

	Pretreatment (−)	Pretreatment (+)	*P* value
***SRK/T***			
ME (D)	0.10 ± 0.53	0.09 ± 0.28	0.828
MAE (D)	0.42 ± 0.33	0.23 ± 0.19	< 0.001*
Refractive surprise (+)	9 (17.3%)	2 (3.8%)	0.024^†^*
Refractive surprise (−)	43 (82.7%)	51 (96.2%)	
***Barrett Universal II***			
ME (D)	−0.06 ± 0.51	−0.01 ± 0.30	0.529
MAE (D)	0.38 ± 0.34	0.24 ± 0.19	0.008*
Refractive surprise (+)	8 (15.4%)	1 (1.9%)	0.016^†^*
Refractive surprise (−)	44 (84.6%)	52 (98.1%)	

Values are presented as mean ± standard deviation

*D* Diopter, *DED* Dry eye disease, *ME* Mean error, *MAE* Mean absolute error

Refractive surprise: MAE ≥ 0.75D; †Chi-squared test; *: statistically significant

In univariate and multivariate logistic regression analysis, the pretreatment of DED significantly lowered the proportion of refractive surprise [univariate: odds ratio (OR) = 0.19/0.11, 95% confidence interval (CI): 0.04–0.91/0.01–0.88, *p* = 0.038/0.038; multivariate: OR = 0.18/0.08, 95% CI: 0.05–0.71/0.01–0.72, *p* = 0.036/0.024 (SRK/T/Barrett Universal II); Table 3]. Age, sex, AL, and K value did not influence the refractive surprises in the univariate and multivariate logistic regression analyses (each *p* > 0.05). In the subgroup analysis in the pretreatment group, pretreatment was more effective on the reduction of refractive surprise in the elderly (> 60 years), men, and patients with a normal range of AL (22–24.99 mm; Table 4). [elderly/men/normal range of AL: *p* = 0.042/0.043/0.027(SRK/T)/0.040/0.018/0.041(Barrett Universal II)]. The pretreatment also showed the effectiveness on the reduction of refractive surprise in the abnormal range of K values with marginally significance. [*p* = 0.063(SRK/T)/0.054(Barrett Universal II)].

**Table 3:** Factors associated with postoperative refractive surprise: univariate and multivariate logistic regression

Variables	SRK/T OR (95% CI, ***p*** value)	Barrett Universal II OR (95% CI, ***p*** value)
***Univariate analysis***		
Age (≤ 60/ > 60)	2.88 (0.35–23.78, 0.327)	2.24 (0.27–18.93, 0.459)
Sex (Men/Women)^a^	1.59 (0.39–6.36, 0.518)	0.69 (0.17–2.72, 0.592)
Pretreatment of DED (No/Yes)	0.19 (0.04–0.91, 0.038)^a^	0.11 (0.01–0.88, 0.038)^a^
AL (22–24.99/< 22 or ≥ 25)	0.94 (0.43–5.42, 0.938)	0.51 (0.06–4.30, 0.533)
Mean K (42–44.99/< 42 or ≥ 45)	1.18 (0.34–4.13, 0.801)	3.05 (0.72–12.94, 0.130)
***Multivariate analysis***		
Age (≤ 60/> 60)	2.49 (0.28–22.43, 0.415)	2.75 (0.26–28.73, 0.397)
Sex (Men/Women)	1.61 (0.37–6.92, 0.524)	0.67 (0.15–3.11, 0.672)
Pretreatment of DED (No/Yes)	0.18 (0.05–0.71, 0.036)^a^	0.08 (0.01–0.72, 0.024)^a^
AL (22–24.99/< 22 or ≥ 25)	0.76 (0.13–4.29, 0.752)	0.30 (0.03–2.99, 0.307)
Mean K (42–44.99/< 42 or ≥ 45)	1.39 (0.37–5.21, 0.628)	4.53 (0.95–21.65, 0.058)

Refractive surprise: MAE ≥ 0.75 D

*AL* Axial length, *CI* Confidence interval, *D* Diopter, *DED* Dry eye disease, *K* Keratometric value, *MAE* Mean absolute error, *OR* Odds ratio

^a^Statistically significant

**Table 4:** Exploring the effects of DED pretreatment in subgroups on the reduction of refractive surprise (multivariate logistic regression)

Variables	SRK/T (95% CI, ***p*** value)	Barrett Universal II (95% CI, ***p*** value)
Age (≤60)	0.22 (0.01–4.71, 0.336)	0.25 (0.02–4.31, 0.345)
Age (> 60)	0.12 (0.02–0.92, 0.042)^a^	0.10 (0.01–0.90, 0.040)^a^
Men	0.25 (0.01–0.93, 0.043)^a^	0.18 (0.01–0.85, 0.018)^a^
Women	0.26 (0.05–1.41, 0.119)	0.21 (0.02–1.95, 0.168)
AL (22–24.99)	0.09 (0.01–0.76, 0.027)^a^	0.11 (0.01–0.91, 0.041)^a^
^a^AL (< 22 or ≥ 25)	3.59 (0.16–81.02, 0.421)	3.96 (0.09–169.11, 0.472)
Mean K (42–44.99)	0.33 (0.06–1.96, 0.224)	0.27 (0.05–1.48, 0.131)
Mean K (< 42 or ≥ 45)	0.18 (0.02–1.18, 0.063)	0.11 (0.01–1.02, 0.054)

Refractive surprise, MAE ≥ 0.75D

*AL* Axial length, *CI* Confidence interval, *D* Diopter, *DED* Dry eye disease, *K* Keratometric value, *MAE* Mean absolute error

^a^Statistically significant

There were no adverse effects, such as elevation of intraocular pressure and ocular infection, in the pretreatment group before and after surgery.

## Discussion

This study showed that short-term treatment of DED prior to cataract surgery improved the accuracy of IOL power calculation. Pretreatment protocols of DED for 2 weeks comprised topical LE and CsA and eyelid scrub with warm compression. In our previous study, we have shown excellent effects of short-term topical corticosteroids in patients with refractory DED [13]. In this study, most patients stated improvement in symptoms after 2 weeks at inpatient clinic. Considering the discomfort of patients having to delay the surgery due to preoperative management and use of topical steroids after the surgery, we set the pretreatment period as 2 weeks. Pretreatment of DED significantly reduced MAE and the number of refractive surprises at 1 month after surgery. Among the several clinical factors including age, sex, AL, and K value, pretreatment of DED before surgery was only related to a reduction in postoperative refractive surprise. Pretreatment was more effective in reducing refractive surprise in the elderly, male, and subjects with a normal range of AL.

The prevalence of DED tends to increase with age [14]. The prevalence is reported from 12.3 to 73.5% worldwide [15, 16], increased from 8.0 to 17.9% in South Korea, where majority of the population includes older individuals [17, 18]. It is known that DED is aggravated after cataract surgery for various reasons, such as excessive light exposure, topical anesthetics and antiseptics, increase in inflammatory cells, and physical damage to the cornea and adjacent structures during operation [19, 20]. Therefore, sufficient management of DED before cataract surgery is recommended to optimize surgical outcomes and improve patient satisfaction [10]. As cataract surgery became more popular with growing needs, the patients’ expectations have increased, and achieving a higher accuracy of IOL power prediction and postoperative DED management are key to meeting these expectations. Many recent studies have proposed the preoperative treatment of DED to prevent postoperative DED aggravation [21], but only a few studies have proposed the importance of preoperative treatment of DED to improve the accuracy of IOL power calculation. Our study showed a meaningful improvement in IOL power prediction by treating DED prior to cataract surgery, using topical LE and CsA and eyelid scrub with warm compression. Currently, the mainline drugs for managing DED are anti-inflammatory agents [22]. LE has been used in various inflammatory ocular diseases, and it exhibits effective anti-inflammatory activity, with fewer events of increased intraocular pressure compared to other corticosteroids [23]. Topical CsA is currently recommended for the treatment of moderate-to-severe inflammation in DED, which downregulates T-cell activity without increasing the risk of infection or immunosuppression [24]. Although CsA improves objective and subjective measures of DED, it requires a longer time to produce a maximal effect [25]. Pflugfelder et al. showed that in a 1-month randomized, double-masked clinical trial, LE showed a significant improvement in central corneal staining, nasal bulbar conjunctival hyperemia, and lid margin injection compared to the control placebo group. In their study, LE was highly effective in the moderate-to-severe DED group, without any common complications such as increase in intraocular pressure [26]. Lee et al. revealed that when LE was used along with eyelid scrubs and warm compression, there were prominent improvements in almost all clinical outcomes in patients with meibomian gland dysfunction (MGD), including an increase in tear breakup time, ocular surface stability, and improved eyelid margin abnormality and a decrease in ocular irritation expressed by the patients [27]. To compensate the relatively long initiating effect of CsA, one study used topical LE with CsA and achieved a faster improvement in DED; it was successful in achieving a rapid relief of symptoms along with a decrease in the common side effect of burning sensation after instillation compared with groups that used artificial tears and CsA together [27]. With the use of topical LE and CsA for 2 weeks prior to cataract surgery, our study significantly improved the accuracy of IOL power prediction. Epitropoulos et al., reported that higher tear osmolarity group showed more variability in average K and anterior corneal astigmatism [11]. Pretreatment with DED seems to increase tear film stability and decrease ocular surface staining, leading to accurate measurement of K values prior to cataract surgery [11].

In our subgroup analysis (Table 4), pretreatment with DED was more effective in men and patients older than 60 years. Prevalence of DED and MGD increased with age and males tended to express less discomfort, suggesting that their DED status could have been masked and underestimated [28, 29]. Furthermore, preoperative treatment of DED was effective in eyes with a normal range of AL (22–24.99 mm). Patients with abnormal range of AL may have a larger error factor compared to those with a normal range of AL, so increasing the accuracy of K value with pretreatment seemed to be more effective in normal AL group.

Detailed screening procedures for DED and selective treatment prior to cataract surgery are definitely beneficial. However, it may be time-consuming and difficult to perform in clinical settings. In fact, more than 80% of preoperative patients showed signs of DED and 75% showed MGD, both of which are well-known factors that aggravate postoperative DED [30, 31]. Hence, we believe that generalized short-term pretreatment of DED could simplify the clinical process in cataract surgery, leading to better outcomes in refractive prediction and postoperative DED. As mentioned earlier, the pretreatment protocol of DED effectively decreased the percentage of refractive surprise in both formulas: from 17.3 to 3.8% (SRK/T) and from 15.4 to 1.9% (Barrett Universal II). In addition, SRK/T formula is prone to induce postoperative refractive error with K value, compared to Barrett Universal II formula. We think that the pretreatment of DED lead more accurate measurement of K value, so leading to higher percentage (65.4 ➔ 94.3%) of MAE ≤ 0.5D especially in SRK/T formula.

This study has several limitations. First, the study was retrospective in nature. Second, the sample size was small. Third, detailed ocular biometry examinations (AL and K) were not evaluated before and after DED pretreatment. Fourth, we did not classify the severity of DED and MGD. Nevertheless, the short-term DED pretreatment group showed a marked improvement in postoperative IOL power prediction compared to the non-pretreatment group. Since this was our first study about effects of the pretreatment of DED on postoperative IOL power accuracy, we wanted to maximize the treatment effects by using topical LE and CsA simultaneously. We have a plan to compare the pretreatment effects of only LE, LE + CsA, and no pretreatment in the future prospective study.

In conclusion, short-term preoperative management of DED using topical LE, CsA and eyelid scrubs with warm compression can improve the accuracy of IOL power calculation. Our pretreatment protocol for DED before cataract surgery can reduce the probability of refractive surprise. Thus, we believe that this protocol may be helpful before cataract surgery, as it is easily applicable and safe, with no adverse effects.

## Data Availability

The datasets used and/or analysed during the current study are available from the corresponding author on reasonable request.
